# Leukotoxin Diols from Ground Corncob Bedding Disrupt Estrous Cyclicity in Rats and Stimulate MCF-7 Breast Cancer Cell Proliferation

**DOI:** 10.1289/ehp.8231

**Published:** 2005-08-08

**Authors:** Barry M. Markaverich, Jan R. Crowley, Mary A. Alejandro, Kevin Shoulars, Nancy Casajuna, Shaila Mani, Andrea Reyna, John Sharp

**Affiliations:** 1 Department of Molecular and Cellular Biology, and; 2 Center for Comparative Medicine, Baylor College of Medicine, Houston, Texas, USA; 3 Department Mass Spectrometry Facility, Washington University Medical School, St. Louis, Missouri, USA

**Keywords:** breast cancer, corncob bedding, endocrine disruptor, estrous cycles, leukotoxin diols

## Abstract

Previous studies in our laboratory demonstrated that high-performance liquid chromatography (HPLC) analysis of ground corncob bedding extracts characterized two components (peak I and peak II) that disrupted endocrine function in male and female rats and stimulated breast and prostate cancer cell proliferation *in vitro* and *in vivo*. The active substances in peak I were identified as an isomeric mixture of 9,12-oxy-10,13-dihydroxyoctadecanoic acid and 10,13-oxy-9,12-dihydroxyoctadecanoic acid, collectively designated tetrahydrofurandiols (THF-diols). Studies presented here describe the purification and identification of the HPLC peak II component as 9,10-dihydroxy-12-octadecenoic acid (leukotoxin diol; LTX-diol), a well-known leukotoxin. A synthetic mixture of LTX-diol and 12,13-dihydroxy-9-octadecenoic acid (isoleukotoxin diol; i-LTX-diol) isomers was separated by HPLC, and each isomer stimulated (*p* < 0.001) MCF-7 cell proliferation in an equivalent fashion. The LTX-diol isomers failed to compete for [^3^H]estradiol binding to the estrogen receptor or nuclear type II sites, even though oral administration of very low doses of these compounds (>> 0.8 mg/kg body weight/day) disrupted estrous cyclicity in female rats. The LTX-diols did not disrupt male sexual behavior, suggesting that sex differences exist in response to these endocrine-disruptive agents.

We recently discovered that housing adult male or female rats on ground corncob bedding blocks male and female sexual behavior and cyclicity (Markaverich et al. 2002b). These results suggested that this bedding material contained endocrine-disruptive substances. We initially postulated that the endocrine-disruptive agent(s) in corncob was likely a phytoestrogen because earlier studies demonstrated that plant isoflavonoids possess estrogenic activity in a variety of experimental systems (Adlercreutz et al. 1992; Bickoff et al. 1958; Markaverich et al. 1995; Martin et al. 1978). On the basis of these observations, we reasoned that the MCF-7 human breast cancer cell proliferation (the E-Screen) assay (Soto et al. 1995) would be a suitable rapid *in vitro* screen for the endocrine disruptors in extracts of ground corncob bedding. Our initial studies on ground corncob bedding extracts led to the purification of two peaks of mitogenic activity on reverse-phase high-performance liquid chromatography (HPLC). Peak I was purified to homogeneity and identified as an isomeric mixture of 9,12-oxy-10,13-dihydroxyocta-decanoic acid and 10,13-oxy-9,12-dihydroxy-octadecanoic acid [tetrahydrofurandiols (THF-diols)] (Markaverich et al. 2002a). The compounds (Figure 1) were synthesized and found to stimulate MCF-7 human breast cancer proliferation *in vitro* and block sexual behavior in male rats (Mani et al. 2005) and female rats and ovarian cyclicity (Mani et al. 2005; Markaverich et al. 2002b) at concentrations approximately 200-fold lower than classical phytoestrogens (Markaverich et al. 1995). In addition, the THF-diols are apparently devoid of estrogenic activity and do not bind to the estrogen receptor (ER) or nuclear type II [^3^H]estradiol binding sites (Markaverich et al. 2002a, 2002b). Thus, the THF-diols were identified as very active endocrine-disruptive agents that block steroid-hormone–dependent pathways through a nonconventional mechanism.

In this article we describe the purification of the peak II component by HPLC and its identification by gas chromatography–mass spectrometry (GC-MS). Synthetic isomeric preparations of the compound stimulated breast cancer cell proliferation and blocked estrous cyclicity in female rats but were devoid of biologic effects on male sexual behavior. Like the THF-diols, this novel endocrine-disruptive agent derived from fatty acid metabolism in plants does not bind to ER or nuclear type II [^3^H]estradiol binding sites and thus antagonizes estrogenic response through non-classical pathways (Maggiolini et al. 2001; Markaverich et al. 1988). The studies emphasize the importance of considering the effects of the environmental housing conditions on experimental model systems and also indicate that human exposure to corncob mitogens with “endocrine-disruptive potential” could represent a significant human health problem.

## Materials and Methods

### Animals and treatment.

We used adult (60-day-old) Sprague-Dawley male and female rats (Harlan Laboratories, Madison, WI) for these studies. Animals were housed in suspended stainless steel wire cages and maintained in compliance with federal guidelines for animal care (Human Health Extension Act of 1985, Public Law 99-158) with appropriate institutional animal care and use committee approval and were treated humanely with regard for alleviation of suffering. Rats were maintained under climate-controlled conditions on a 12-hr light/dark cycle (lights on at 0600 hr) with food (Harlan Teklad Global Diet no. 2014 containing no alfalfa, soybean, or phytoestrogen components; Harlan Teklad, Madison, WI) and water provided *ad libitum*. Male and female rats were acclimated to this environment for at least 3 weeks before the initiation of the studies.

**Figure 1:**
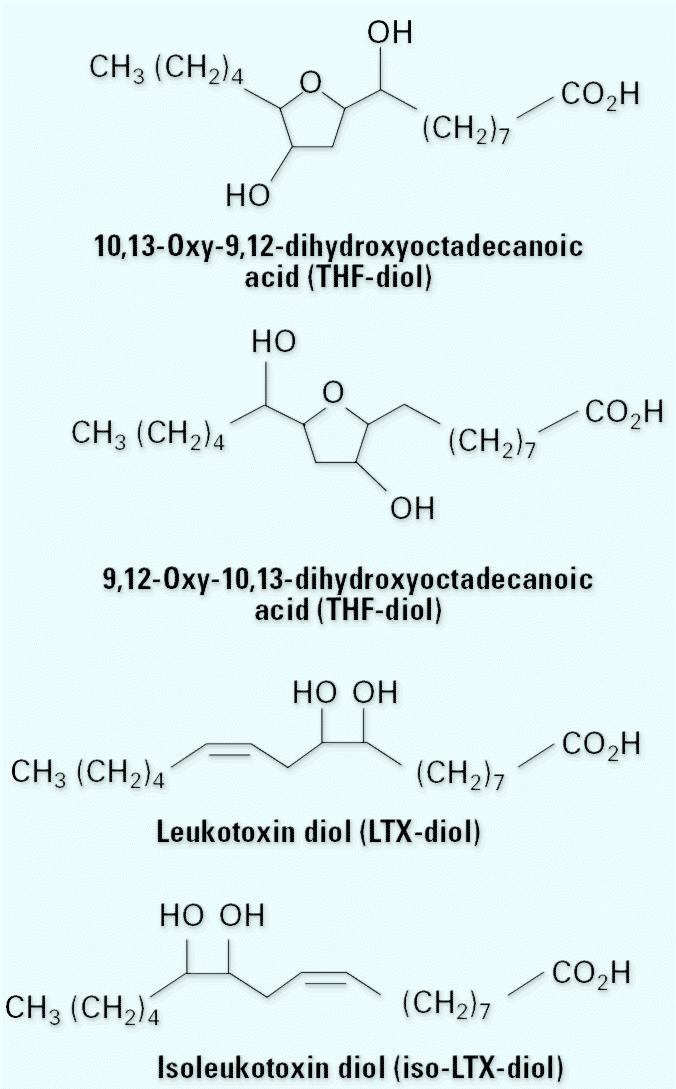
Structures of LTX-diols and THF-diols.

For the cycling studies, daily vaginal smears were collected from eight adult female rats housed under standard conditions and given tap water containing 2% Tween 80 vehicle as a drinking solution for 30 days to establish baseline controls and confirm that the animals were cycling in a normal fashion. This vehicle has no significant effects on ovarian cyclicity in rats or on male or female sexual behavior relative to tap water controls (Mani et al. 2005; Markaverich et al. 1988, 2002a, 2002b). On day 31, the cycling female rats were given a 1:1 mixture of the 9,10-dihy-droxy-12-octadecenoic acid [leukotoxin diol (LTX-diol)] isomers (2 μg/mL) in the tap water–Tween 80 vehicle for an additional 30 days. This dose was chosen on the basis of previous published studies with the THF-diols, which are structurally similar to the LTX-diols (Figure 1) and which we suspected would have similar biologic activities. Daily vaginal smears were collected throughout the 30-day treatment period and evaluated for cyclicity as previously described (Markaverich et al. 2002a, 2002b). In this particular study, the female rats served as their own controls because their ovarian cycles were determined before and after LTX-diol treatment. Previous studies with the THF-diols demonstrated that equivalent results are obtained with separate groups of controls or treated animals or with animals serving as their own controls (Markaverich et al. 2002a, 2002b).

To determine whether the LTX-diols affect male reproductive function, three replicate studies employing six established adult male Sprague-Dawley breeder rats were performed as previously reported for the THF-diols (Mani et al. 2005; Markaverich et al. 1988, 2002a, 2002b). The male breeder rats who had side litters at least three times were housed under reversed lighting conditions in hanging wire cages and were provided a drinking solution containing LTX-diol isomers (2μg/mL of drinking solution) for 1–4 weeks. Sexual behavior was evaluated by examining the number of mounts, intromissions, ejaculations, ejaculation latencies (in seconds), and grooming frequencies in a 30-min test period with each sexually receptive female as per well-established procedures in our laboratories (Hull et al. 1990). Ovariectomized steroid-primed Sprague-Dawley female rats were used as stimulus animals and were brought into behavioral estrus by a subcutaneous priming injection of 2 μg estradiol benzoate in sesame oil 48 hr before receiving 100 μg progesterone (subcutaneously), as previously described (Markaverich et al. 2002b).

### Reagents and solvents.

We obtained linoleic acid and *m*-chloroperoxybenzoic acid (mCPBA) from Sigma Chemical (St. Louis, MO). *N*,*O*,-bis(trimethylsilyl)trifluoro-acetamide with 10% trimethyl chlorosilane (BSTFA) was obtained from Pierce Chemical (Rockford, IL). We purchased Sep-Pak C_18_ cartridges (3 cc) from Waters Corporation (Milford, MA), and C_18_ minicolumns from Varian (Walnut Creek, CA). All solvents were HPLC grade from Burdick and Jackson (Muskegon, MI).

### Purification of peak II from corncob bedding.

We prepared the ethyl acetate extract of ground corncob bedding as previously described (Markaverich et al. 2002a, 2002b). The dried extract was redissolved in approximately 20 mL HPLC-grade methanol, and 2 mL aliquots were analyzed on a Beckman Gradient HPLC System (Beckman Coulter, Fullerton, CA) equipped with a diode array detector and a Dynamax Ultrasphere-Octyl HPLC column (Varian). The column was equilibrated in acetonitrile (CH_3_CN):water (30:70) containing 0.1% acetic acid at a flow rate of 4 mL/min. A linear gradient to 40% CH_3_CN was initiated 5 min after injection and completed by 90 min. Fractions (1 min) were collected, and triplicate aliquots were assayed for mitogenic activity in MCF-7 breast cancer cells.

### Mitogenic effects of HPLC fractions and synthetic LTX-diol on MCF-7 human breast cancer cells.

**Figure 2:**
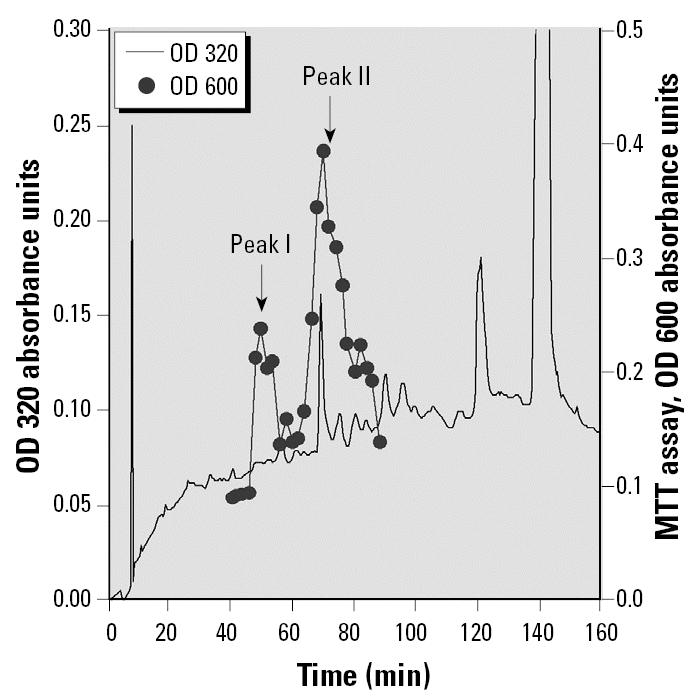
Reverse-phase HPLC of mitogenic activity from ground corncob bedding. An aliquot (2 mL) of an ethyl acetate extract of ground corncob bedding was injected onto a Dynamax C8 column eluted as described in “Materials and Methods.” and 1-minute fractions were collected. Aliquots of the fractions were dried and assayed for mitogenic activity in cultured MCF-7 human breast cancer cells. The peak of mitogenic activity at approximately 45 min (peak I) was previously identified as THF-diols, and the peak of activity at approximately 70 min (peak II) was collected and analyzed by GC-MS.

For the assessment of mitogenic activities of the various preparations on MCF-7 cells, we followed previously described methods (Markaverich et al. 2002a, 2002b). Briefly, we added the aliquots of the HPLC fractions (1–5 μL) or HPLC-purified synthetic LTX-diol isomers in redistilled ethanol to cultured MCF-7 cells grown in phenol red–free Dulbecco’s modified Eagle’s medium (DMEM) containing 5% charcoal-stripped, sulfatase-treated fetal calf serum. Cell number was determined 7 days after treatment by hemocytometer counts or by the MTT (methyl thiazoyl tetrazolium) absorbance assay (Markaverich et al. 2002a). HPLC fractions containing peak II of mitogenic activity (Figure 2) were collected on C_18_ mini-columns (Varian, Lake Forest, CA) and analyzed by GC-MS. The mass spectra of the TMS (trimethylsilylether) derivatives of the two isomers were in agreement with those reported in the literature (Halankar et al. 1989). Synthetic LTX-diol and 12,13-dihy-droxy-9-octadecenoic acid (iso-LTX-diol) were redissolved in 100% redistilled ethanol and added to the cultured cells.

### GC-MS studies.

For GC-MS analysis, the HPLC peak components or authentic compounds were derivatized by a number of procedures. We prepared the trimethylsilyl ethers by adding a 1:4 mixture of BSTFA:CH_3_CN to the dried samples, which were vortexed and heated at 70°C for 30 min. Methyl esters of the carboxylic acids in the HPLC fractions or synthetic compounds were prepared by redissolving the dried samples in methanol:1 N HCl (1:3) and heating at 70°C for 60 min. The samples were taken to dryness under nitrogen and redissolved in methanol before analysis. We performed catalytic hydrogenation to reduce double bonds in the unknowns or synthetic compounds by redissolving the dried samples in 1 mL methanol in the presence of a small amount of platinum while bubbling H_2_ gas slowly into the sample to evaporate the methanol. The dried material was redissolved in methanol and centrifuged to remove the platinum pellet. The sample was transferred to a clean vial and taken to dryness under nitrogen, and the TMS derivative was prepared as described above.

The derivatized samples were analyzed on a Varian 3400 gas chromatograph interfaced with a Finnigan SSQ 7000 mass spectrometer (ThermoFinnigan, San Jose, CA) equipped with a DB-1 column (12.5 m, 0.2 mm inner diameter, 0.0.33 μm film coating; P.J. Cobert, St. Louis, MO). The initial temperature of 120°C was held for 1 min after sample injection, increased linearly to 270°C at 10°C/min, and held at 270°C for 5 min. For electron ionization, the source temperature, electron energy, and emission current were 200°C, 100 eV, and 300 μA, respectively. For chemical ionization, the source temperature, electron energy, and emission current were 140°C, 240 eV, and 300 μA, respectively. Methane was used as carrier gas for chemical ionization. The injector and transfer line temperatures were 250°C.

### Synthesis of 9,10-epoxy-12-octadecenoic and 12,13-epoxy-9-octadecenoic acids.

The epoxy acids were synthesized from linoleic acid as previously described (Zheng et al. 2001), with slight modifications. Linoleic acid (10 mg) was dissolved in 3 mL methylene chloride. mCPBA (70 mg) was added to the sample, and the mixture was stirred at 22°C for 5 hr. The reaction mixture was washed sequentially with 1 mL portions of 25 mM NH_4_HCO_3_, saturated saline solution, and H_2_O. The washed extract was evaporated to dryness under N_2_, and the waxy white solid was stored at –20°C.

### Synthesis and purification of LTX-diol and iso-LTX-diol.

Approximately 20 mg of the waxy white solid described above was dissolved in THF:H_2_O:5% perchloric acid (5:1:1) and stirred at 22°C for 1 hr. The products were extracted into ethyl acetate and evaporated to dryness under vacuum. LTX-diol and iso-LTX-diol were separated from linoleic acid and THF-diols in these synthetic mixtures by chromatography on C_18_ reverse-phase mini-columns. To accomplish the separation, the dry synthetic mixture was redissolved in approximately 300 μL CH_3_CN. This solution was applied to a C_18_ minicolumn pre-equilibrated with 2 mL CH_3_CN and 4 mL 0.5% acetic acid. The column was washed with 40% CH_3_CN in 0.5% acetic acid to elute residual by-products of the mCPBA. The column was then eluted with 50% CH_3_CN in 0.5% acetic acid to obtain the THF-diols. Elution with 60% CH_3_CN/0.5% acetic acid facilitated the collection of an isomeric mixture of LTX-diol and iso-LTX-diol. Linoleic acid elutes from the C_18_ minicolumns with higher concentrations (> 70%) of CH_3_CN and therefore was removed from these LTX-diol preparations. The identity and purity of the eluents and contaminants in each of the above fractions were confirmed by GC-MS.

### HPLC separation of LTX-diol and iso-LTX-diol.

To compare the biologic activities of the two isomers, separation of LTX-diol and iso-LTX-diol was performed on a Beckman HPLC system equipped with an Altex Ultrasphere-ODS semipreparative column (10 mm × 25 cm) (Beckman Coulter) eluted isocratically with 45% CH_3_CN containing 0.1% acetic acid at a flow rate of 2.0 mL/min. Compounds were detected at 203 nm with a Beckman diode array detector. The peaks at 32 and 36 min were confirmed by GC-MS to be pure iso-LTX-diol and LTX-diol, respectively.

### Effects of LTX-diol and iso-LTX-diol on MCF-7 breast cancer cell proliferation.

The LTX-diol isomers isolated by HPLC were taken to dryness under nitrogen at 50°C, weighed, and redissolved at known concentrations in 100% ethanol for the cell proliferation assays as described above. Briefly, MCF-7 cells grown as described above were treated with a range of LTX-diol or iso-LTX-diol concentrations (0.1–10 μg/mL), and cell number was determined 7 days after treatment, as described previously (Markaverich et al. 2002a, 2002b). The isomers had equivalent biologic activity in the MCF-7 cell proliferation assay; therefore, the mixture was used for the remaining studies.

### LTX-diol competition for [^3^H]estradiol binding to ER and type II sites in rat uterine nuclear fractions.

Earlier studies on partially purified ground corncob extracts containing the mitogenic activity revealed that these preparations did not compete for [^3^H]estradiol binding to ER or nuclear type II [^3^H]estradiol binding sites. This was later confirmed with pure preparations of synthetic THF-diol (Markaverich et al. 2002a, 2002b). Thus, we suspected that the biologic effects of the LTX-diols were probably not mediated via direct interactions with either ER or the nuclear type II sites because the LTX-diols are structurally related to the THF-diols. To directly evaluate these possibilities, we assessed LTX-diol competition for [^3^H]estradiol binding to ER or type II sites (Markaverich et al. 2002a, 2002b). Briefly, adult female Sprague-Dawley rats were ovariectomized and implanted with 20-μg estradiol-containing beeswax pellets to promote nuclear ER retention and stimulate nuclear type II sites (Markaverich et al. 2002b). Seven days after treatment, the uteri were removed from these animals, stripped of extraneous tissues, weighed, and chilled in ice-cold saline before analysis. For ER competition studies, rat uterine tissue was homogenized in 10 mM Tris, 1.5 mM EDTA, and 0.1 mM dithiothreitol, pH 7.4 at 22°C, and uterine nuclear suspensions were incubated at 37°C for 30 min in the presence of 10 nM [^3^H]estradiol ± 0.01–10 μM LTX-diol or diethylstilbestrol (DES) under conditions that measure only [^3^H]estradiol binding to ERs (Markaverich et al. 1981). For type II site competition studies, uterine tissue was homogenized in TE buffer (10 mM Tris, 1.5 mM EDTA) and nuclear suspensions were incubated at 4°C × 60 min in the presence of 30 nM [^3^H]estradiol ± 0.00015–30 μM LTX-diol or luteolin (a competitive inhibitor of type II sites but not ERs) under conditions that measure [^3^H]estradiol binding to type II sites but not occupied ERs. After incubation, the nuclear suspensions for ER or nuclear type II site assays were washed 3 times by resuspension and centrifugation (800 × *g* × 7 min) in 1 mL TE buffer, and the final washed pellets were extracted with 1 mL 100% ethanol. Radioactivity in the ethanol extract was determined by liquid scintillation spectrometry (Markaverich et al. 1981). [^3^H]Estradiol binding in the absence of competitor (controls) was approximately 10,000 cpm for ERs and 30,000 cpm for type II sites. Results were expressed as the percentage of [^3^H]estradiol bound in the presence of the indicated concentrations of LTX-diol, DES, or luteolin relative to the vehicle control (100%).

### Statistical analyses.

We analyzed data from cell proliferation assays and animal cycling studies (body weights, fluid consumption) statistically by analysis of variance (ANOVA) and Tukey’s test on the treatment means using InStat (GraphPad Software Inc., San Diego, CA). The data recorded from the behavioral tests for male sexual behavior (data not shown) were compared using Kruskal-Wallis ANOVA followed by Dunn’s method for post hoc comparison using Graph Pad Prism software, version 4 (Graph Pad Software Inc.).

## Results

### Purification of mitogenic agents in ground corncob extract.

**Figure 3:**
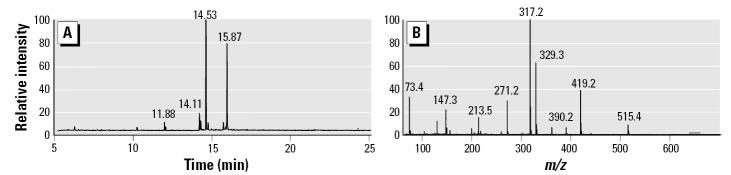
GC-MS analysis of HPLC peak II component isolated from ground corncob. An aliquot of the pooled peak II fractions (Figure 1) was derivatized with BSTFA to generate the trimethylsilyl ether and chromatographed on a DB-1 column as described in “Materials and Methods.“ (*A*) Total ion chromatogram for this sample. (*B*) Electron ionization spectrum for peak at 14.53 min.

HPLC analysis of an ethyl acetate extract of ground corncob animal bedding separated two major peaks of mitogenic activity (peaks I and II), as shown in Figure 2. Peak I was previously identified as a mixture of the THF-diol isomers (Figure 1) shown to disrupt male and female endocrine function *in vivo* (Mani et al. 2005; Markaverich et al. 2002a, 2002b). We purified and identified peak II in the present study. Preliminary studies revealed that the peak II component did not contain compounds sufficiently volatile for GC-MS without derivatization. Therefore, the material was derivatized with BSTFA and analyzed by GC-MS. Figure 3A represents the total ion chromatogram obtained by GC-MS analysis showing two major peaks that eluted from the column. When run in the electron ionization mode, the first peak at 14.53 min generated the spectra shown in Figure 3B. Positive chemical ionization data (not shown) determined that the molecular weight of the BSTFA-unknown derivative was 530 amu. Catalytic hydrogenation indicated the presence of one carbon–carbon double bond. Reaction with methanolic HCl before derivatization with BSTFA produced a new peak with a molecular weight consistent with the replacement of one TMS group with a methyl group. This observation indicated the presence of a COOH group in the unknown because carboxylic acids are readily esterified in either methanolic HCl or BSTFA. Further inspection of the spectral data suggested that the compound was a 9,10-diol derived from linoleic acid. This compound would have a molecular weight of 530 amu when derivatized with BSTFA and would be expected to fragment between the 9 and 10 positions (Murphy 1993), leading to fragments at *m*/*z* 317 and 213, and between carbons 10 and 11, producing *m*/*z* 419. Loss of 90 amu [–Si(CH_3_)_3_OH)] from the *m*/*z* 419 accounts for the *m*/*z* 329 result. The unknown in Figure 3B was tentatively identified as LTX-diol. This was confirmed with a match of spectra in the literature, and the compound has a molecular weight of 314 amu. (Draper and Hammock 2000; Sugiyama et al. 1987).

### Synthesis and purification of LTX-diol and iso-LTX-diol.

**Figure 4:**
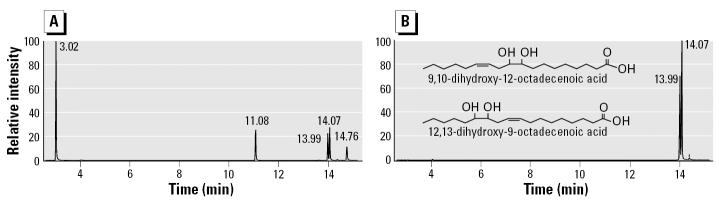
GC-MS analysis of synthetic LTX-diol. The LTX-diol mixture, synthesized as described in “Materials and Methods,” was derivatized and analyzed as described in Figure 2. (*A*) Total ion chromatogram of the sample before cleanup on C_18_ reverse-phase resin. (*B*) Total ion chromatogram after cleanup on the C_18_ reverse-phase resin.

**Figure 5:**
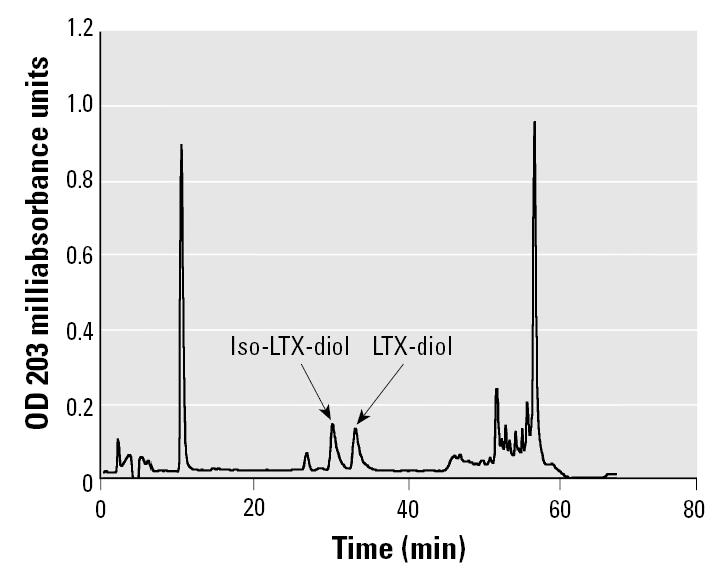
Separation of LTX-diol isomers by reverse-phase HPLC. The synthetic mixture of LTX-diol and iso-LTX-diol shown in Figure 3 was separated and detected as described in “Materials and Methods.”

For confirmation of structure and biologic activity, we synthesized an isomeric LTX-diol mixture as described in “Materials and Methods.” This procedure involved forming the epoxides from linoleic acid and then opening the epoxide ring to generate the vicinal diols. A diepoxide is also formed that will cyclize and generate the THF-diol that we identified from corncob bedding (Mani et al. 2005; Markaverich et al. 2002a, 2002b). Unreacted linoleic acid and by-products of mCPBA not removed by extraction were all present in the reaction mixture containing LTX-diol and iso-LTX-diol (Figure 4A; peaks at 13.99 and 14.07 min). Solid-phase extraction on reverse-phase C_18_ cartridges (Waters Corp.) removed the straight-chain hydroxy fatty acids and separated LTX-diol and iso-LTX-diol from the other components (Figure 4B). Thus, we were able to purify an LTX-diol and iso-LTX-diol isomer mixture to near homogeneity by this procedure. The LTX-diol and iso-LTX-diol isomers were separated by HPLC (Figure 5), and the individual isomers were used for the cell proliferation assays.

### Effects of synthetic LTX-diol and iso-LTX-diol on MCF-7 cell proliferation.

**Figure 6:**
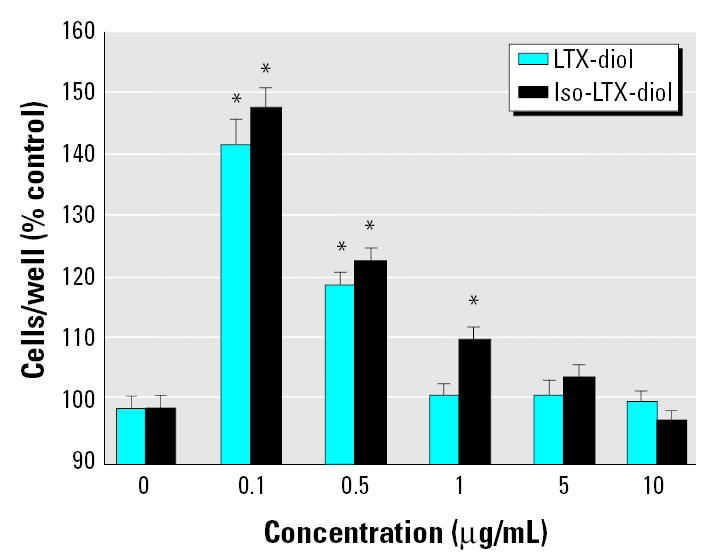
Effects of the LTX-diol isomers on MCF-7 human breast cancer cells grown for 7 days in the absence or presence of of LTX-diol or iso-LTX-diol. Cell number was determined by hemocytometer counts. *Significantly different from controls or other LTX-diol dose levels (*p* < 0.001). Data represent the mean ± SEM for at least 12 determinations.

LTX-diol and iso-LTX-diol purified by HPLC (Figure 5) were added to tissue culture medium in 2 μL ethanol such that the final concentration varied from 0.1 to 10 μg/mol (0.32–1.6 μM; Figure 6). Both isomers stimulated MCF-7 human breast cancer cell proliferation to equivalent degrees at concentrations ranging from 0.32 to 1.6 μM. These concentrations of the LTX-diols are > 400-fold lower than those doses generally characterized as toxic in insect cell cultures *in vitro* (Hayakawa et al. 1986; Sisemore et al. 2001). Thus, at these lower concentrations, the LTX-diols are mitogenic, and we anticipate that the compounds will stimulate the proliferation of hormone-dependent and hormone-independent breast and prostate cancer cells if their mechanism of action is similar to that of the THF-diols (Mani et al. 2005; Markaverich et al. 2002a, 2002b). Although LTX-diol identified as the peak II component isolated from ground corn-cob in this study is an auto-oxidation product of linoleic acid in animals, a cytosolic epoxide hydrolase can metabolize the epoxy fatty esters to their vicinol diols (Halankar et al. 1989). This same enzyme exists in plants, as well (Blee and Shuber 1992), and therefore, it is feasible that epoxy fatty acids, known to be abundant in the plant kingdom, may be metabolized to vicinol diols.

### LTX-diol competition for ER and nuclear type II sites.

**Figure 7:**
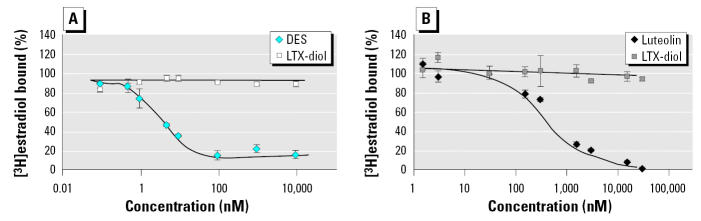
Synthetic LTX-diol competition for ER (*A*) and nuclear type II sites (*B*), as described in “Materials and Methods.”

Data in Figure 7 show that the LTX-diol mixture failed to compete for [^3^H]estradiol binding to rat uterine nuclear ER (Figure 7A) or nuclear type II sites (Figure 7B). These data are consistent with results obtained with partially purified ground corncob extracts containing both the THF-diols and LTX-diols (Markaverich et al. 2002a, 2002b). Thus, it is unlikely that the effects of these compounds on cellular proliferation or biologic response *in vivo* in male or female rats involve binding interactions with either ER or type II sites.

### Effects of LTX-diols on the estrous cycle of female rats and male sexual behavior.

**Figure 8:**
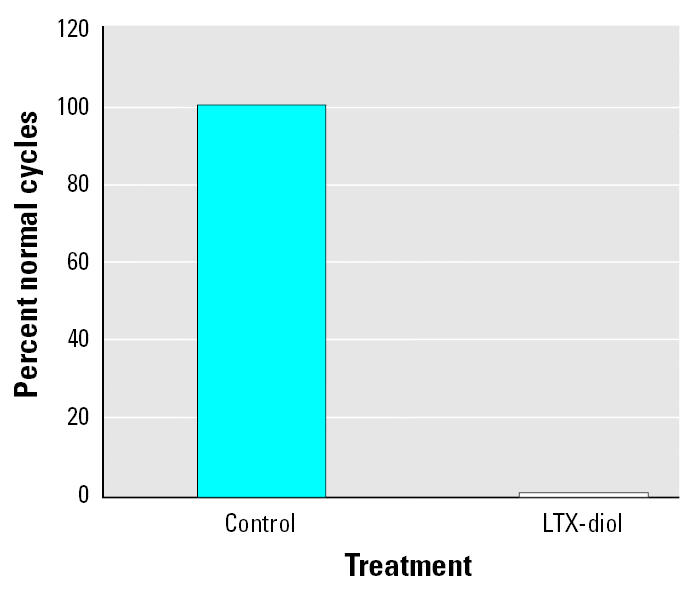
Effects of LTX-diols on the estrous cycle of adult female rats (*n* = 8). See “Materials and Methods” for details.

Adult female rats displayed normal 4.6 ± 0.25 day estrous cycles for 30 days when maintained on tap water/5.0% Tween 80 vehicle (Figure 8). Administration of LTX-diols to these animals at a daily dose level of >> 0.8 mg/kg body weight (2 μg/mL drinking solution) for 30 days disrupted the estrous cycle in 100% of the animals (Figure 8); this disruption was evident within the first 3 or 4 days after treatment and was sustained for the 30-day treatment period. These animals displayed vaginal smears resembling atypical sustained metestrus as described for THF-diols (Markaverich et al. 2002a, 2002b). Consequently, these atypical smears were not appropriate for differential cell count analyses to define a potential mechanism of action of these compounds; we are currently performing detailed mechanistic studies with these compounds *in vitro*. We found no significant treatment effects on body weights (controls = 265 ± 8.7 g; LTX-diol = 243 ± 4.8 g) or fluid consumption (controls = 38.85 mL/day/rat; LTX-diol = 38.75 mL/day/rat). Based on fluid consumption, the LTX-diol–treated female rats consumed >> 0.7 ± 0.072 mg LTX-diol/kg body weight per day. This higher dose level than that used for the THF-diols (>> 0.3 mg/kg body weight per day) was due to the difference in fluid volume consumption by the animals in the two studies (Markaverich et al. 2002a, 2002b).

Treatment of established breeder males with the LTX-diol drinking solution for 1–4 weeks had no significant effect on mounting, intromission, ejaculation, ejaculation latency, or grooming behavior (data not shown). These results are in sharp contrast to studies with similar doses of THF-diols, which completely blocked male sexual behavior (Mani et al. 2005; Markaverich et al. 2002a, 2002b). These findings suggest that sex differences may exist in responses to the THF-diols and the LTX-diols, or that higher doses of the LTX-diols are required to block male sexual behavior. More definitive dose–response and time studies to address these possibilities are in progress. Although we have not directly quantified the levels of LTX-diol or THF-diol in corncob bedding by GC-MS, it is clear from HPLC analysis of a number of corncob extracts that the concentration of these disruptor/mitogenic agents in different lots or varieties of corncob, and genetically engineered corn, vary significantly (Markaverich BM, Alejandro MA, Crowley JR, unpublished data). Thus, these differences in the relative levels of these compounds in ground corncob may serve to limit or enhance exposure and potential toxicity.

## Discussion

Previous studies from our laboratories described the presence of an endocrine disruptor in ground corncob bedding that blocked male and female sexual behavior and cyclicity (Mani et al. 2005; Markaverich et al. 2002a, 2002b) in rats. The endocrine-disruptive activity copurified on HPLC with two peaks of mitogenic activity, leading us to believe that the mitogenic agents and endocrine disruptors were the same compounds. We identified the first mitogenic HPLC peak component(s) as the THF-diols (Figure 1). These compounds were synthesized and shown to stimulate MCF-7 breast cancer cell proliferation *in vitro* and block reproductive behavior and cyclicity in rats at doses in the range of 0.35–0.7 mg/kg body weight (Mani et al. 2005; Markaverich et al. 2002a, 2002b). In the present study we identified a second pair of mitogenic agents (LTX-diols) in HPLC peak II (Figure 2) from corncob bedding that also disrupt the estrous cycle of rats (Figure 8). The LTX-diols maximally stimulate MCF-7 human breast cancer cell proliferation at doses (0.1 μg/mL) that are 50-fold lower than those of the THF-diols (5 μg/mL), suggesting that the compounds have significantly different mitogenic activities (Markaverich et al. 2002a). The LTX-diols were approximately equivalent to THF-diols [0.3 mg/kg/day (Markaverich et al. 2002a)] in terms of endocrine-disruptive activities. When female rats were given the vehicle for 30 days, they displayed normal estrous cycles (4.6 ± 0.25 days); however, consumption of >> 0.7 mg/kg LTX-diol and iso-LTX-diol disrupted cyclicity within 3 or 4 days, and this suppression was sustained throughout the 30-day treatment period. Vaginal smears taken from the LTX-diol–treated animals indicated that a prolonged atypical metestrous state was induced, as previously observed for the THF-diols (Mani et al. 2005; Markaverich et al. 2002a, 2002b). No significant treatment effects on fluid consumption or body weights of the animals were observed, suggesting that systemic toxicity was not a problem.

The doses of LTX-diols required to block estrus in the present studies were equivalent to the dose level of THF-diols administered to adult male and female rats (0.35–0.75 mg/kg body weight) to block sexual behavior and cyclicity (Mani et al. 2005; Markaverich et al. 2002a, 2002b). We suspect the LTX-diols and THF-diols are acting through similar mechanisms. The actual concentration of THF-diols and LTX-diols administered in all of these studies was 2 μg/mL of the Tween 80 drinking solution. The difference in daily doses delivered to the male and female rats in the various studies is attributed to differences in body weights and fluid consumption volumes. Because we observed a virtual 100% block of estrous cyclicity by LTX-diols in the present studies, and of male and female sexual behavior and cyclicity in response to the THF-diols in previous studies (Mani et al. 2005; Markaverich et al. 2002a, 2002b), the actual concentration of these compounds required to block biologic response may be lower than that used here. Once we have sufficient quantities of the compounds on hand, extensive dose studies will be completed with the individual compounds alone and combined to determine whether synergistic interactions exist.

Surprisingly, the LTX-diol isomers did not block male sexual behavior in the present studies. This is in marked contrast to data obtained with the THF-diols (Mani et al. 2005). At present, we have no explanation for this discrepancy other than that there may be dose or sex differences with respect to the response to the LTX-diols and/or differences in the biologic fate of these compounds. It is clear that both the LTX-diols (Figure 8) and THF-diols (Markaverich et al. 2002a) block estrous cyclicity. Housing adult female rats on corncob bedding blocked female sexual behavior (lordosis), but we have not evaluated the effects of the LTX-diols on this parameter. Studies are under way to delineate effects of the LTX-diols and THF-diols on male and female sexual behavior and to define the biologic fate and mechanism of action of these compounds in both sexes. The THF-diols are very closely related to the LTX-diols (both likely evolving from linoleic acid pathways), and we suspect that these compounds are modulating gonadotrophic-hormone–releasing hormone (GHRH) release through nitrous oxide (NO)–dependent pathways. Leukotoxins stimulate NO release from mammalian cells (Ishizaki et al. 1995), and NO mediates male and female sexual behavior (Hull et al. 1994; Ishizaki et al. 1995) via stimulating GHRH release from hypothalamic neurons (Moses 1999). Therefore, it is possible that the THF-diols and LTX-diols disrupt endocrine function via hypothalamic pathways involving GHRH release. We are currently exploring these possibilities.

Similarly, we do not yet understand the mechanisms underlying the mitogenic activities of the THF-diols or LTX-diols in human cancer cells *in vitro* or *in vivo*. However, *in vivo* pathways may involve the modulation of hypo-thalamic–pituitary–gonadal axes and lipid metabolism as well. If the THF-diols or LTX-diols modulate luteinizing-hormone–releasing hormone release via pathways related to NO synthase, phospholipase A (PLA), cyclooxygenase (COX), or lipoxygenase (LOX), the compounds may directly or indirectly control breast or prostate cancer growth and proliferation via modulation of gonadotropin release and/or ovarian or testicular steroidogenesis. Effects on male and female sexual behavior and cyclicity are certainly consistent with this notion (Mani et al. 2005; Markaverich et al. 2002a, 2002b). In addition, although the THF-diols and LTX-diols on ground corncob extracts stimulate estrogen-dependent (MCF-7 cells) cell proliferation, the compounds also stimulate cell proliferation in estrogen-independent breast cancer (MDA-MD-231 cells) and prostate cancer (LNCap vs. PC-3 cells) cell lines (Markaverich et al. 2002a, 2002b) *in vitro*. These findings imply direct effects on nonestrogenic cellular pathways controlling cell proliferation, as well. An association also exists between COX activity and prostaglandin E_2_ induction of aromatase in MCF-7 breast cancer cells. Thus, the potential exists for generating estrogens required for cell proliferation (Brueggemeir et al. 2001; Richards et al. 2002). Because THF-diols and/or LTX-diols may affect COX pathways involved in prostaglandin synthesis, it is also possible that these compounds modulate aromatase. Tetradecanoyl phorbol acetate (TPA) induction of COX activity in MDA-MB-231 cells results in enhanced proliferation (Brueggemeir et al. 2001; Richards et al. 2002), and the THF-diols and /or LTX-diols may also control the proliferation of ER-negative cells by modulating COX activity. The dose–response data in Figure 5 are certainly consistent with the regulation of enzymatic pathways, where lower doses might be expected to stimulate enzymatic activity and higher concentrations may inhibit enzymatic activity via substrate inhibition. Alternatively, there may be intracellular receptors for the THF-diols and LTX-diols that would explain their mitogenic activities at lower concentrations and inhibitory properties at higher concentrations (Markaverich et al. 2002a, 2002b). Classical bell-shaped dose–response curves are typically seen for compounds such as estradiol that mediate their effects via ER binding mechanisms (Clark and Peck 1979). Thus, it is certainly possible that intracellular receptors exist for the THF-diols and/or LTX-diols, and we will be evaluating these possibilities.

Epidermal growth factor (EGF) stimulation of cell proliferation involves membrane-associated PLA-mediated release of arachidonic acid and linoleic acids from the cell membrane. The conversion of these fatty acids to prostaglandins (Nolan et al. 1988) or linoleic acid metabolites [9-hydroxyoctadecadienoic acid (9-HODE), 12-HODE, and 13-HODE] mediates EGF stimulation of [^3^H]thymidine incorporation into DNA (Glasgow and Eling 1990, 1994), cell cycle transition, and apoptosis (Durgam and Fernandes 1997; Kachhap et al. 2000; Tong et al. 2002). Breast cancer specimens contain higher concentrations of PLA than do benign breast tissues, and low PLA activity is associated with longer disease-free interval and survival even though no relationship was noted between PLA and ER or progesterone receptor status (J. Yamashita et al. 1995; S. Yamashita et al. 1993, 1994). In MCF-7, MCF-10, and MDA-MB-231 human breast cancer cells, LOX, but not COX, inhibitors block EGF/transforming growth factor α stimulation of 12-HODE, and 13-HODE production and cellular proliferation (Natajaran et al. 1997; Reddy et al. 1997). A number of LOX inhibitors including nordihydroguaiaretic acid, baicalein, and Rev-5901 inhibit MCF-7 and MDA-MB-231 cell proliferation and induce apoptosis. LOX products [5-eicosatrienoic acid (5-HETE), 12-HETE] reverse these effects (Natajaran et al. 1997). EGF stimulation of MCF-7 cell proliferation causes a dose-dependent increase in the formation of LOX products, including 12-HETE (Tong et al. 2002). Thus, LOX products (HODEs, HETEs) stimulate proliferation of these cells. THF-diols and LTX-diols are derived from linoleic acid pathways that generate HODEs and HETEs. It is possible that the THF-diols and LTX-diols modulate cellular proliferation by controlling the synthesis of these linoleic acid metabolites and/or by mimicking these compounds as mitogenic agents.

In addition to their effects on endocrine-regulated pathways and tumor cell proliferation (Mani et al. 2005; Markaverich et al. 2002a, 2002b), exposure to THF-diols and LTX-diols may cause additional health problems. Hydroxy fatty acids are substrates for liver glucuronosyltransferases (Jude et al. 2001; Street et al. 1996). Although this pathway is a probable means of detoxification of dihydroxy fatty acids, compounds such as LTX-diol are subject to inhalation and may be trapped in the lung as the free hydroxy fatty acids. Farmers are at high risk for respiratory problems and account for 30% of adults suffering from respiratory illness. Risk factors are believed to include exposure to fungus, molds, pesticides, organic dust from a variety of areas including animal bedding, and silicosis (Kansas State University Cooperative Extension Service 1981; North Carolina Cooperative Extension Service 1995). Uncharacterized chemical agents in these materials may include the LTX-diols and THF-diols. In addition to being used as bedding for small animals, ground or milled corncob is also used as adsorbent for chemical spills, a polishing agent for metals, and a pesticide carrier for insects such as spider mites and fire ants. This product is also used as cat litter. Thus, exposure of the general public to toxic agents in ground corncob is likely. Clearly, these fatty acid diols stimulate breast cancer cell proliferation *in vitro* and disrupt reproductive function in rats at relatively low concentrations. Sustained exposure to such compounds may represent a significant health hazard.
